# Proliferative Glomerulonephritis With Monoclonal IgG Deposits: Another Unusual Presentation

**DOI:** 10.7759/cureus.56957

**Published:** 2024-03-26

**Authors:** Pranjal Sharma, Carmen Julius, Herlitz Leal, Ripudaman S Munjal, Gagandeep Dhillon, Ram K Verma, Harpreet Grewal, Venkata S Buddhavarapu, Rahul Kashyap

**Affiliations:** 1 Clinical Research, Global Remote Research Program, St. Paul, USA; 2 Nephrology, Premier Renal Care Associates, Cuyahoga Falls, USA; 3 Internal Medicine/Nephrology, Northeast Ohio Medical University, Rootstown, USA; 4 Pathology, Akron Children's Hospital, Akron, USA; 5 Pathology, Cleveland Clinic, Cleveland, USA; 6 Nephrology, Kaiser Permanente, Stockton, USA; 7 Physician Executive Master of Business Administration, University of Tennessee, Knoxville, USA; 8 Internal Medicine, University of Maryland Medical Center, Baltimore, USA; 9 Sleep Medicine, Parkview Health System, Fort Wayne, USA; 10 Radiology, Florida State University College of Medicine, Pensacola, USA; 11 Hospital Medicine, Banner Health, Phoenix, USA; 12 Medicine, Drexel University College of Medicine, Philadelphia, USA; 13 Global Clinical Scholars Research Training, Harvard Medical School, Boston, USA; 14 Research, Global Remote Research Program, St. Paul, USA; 15 Critical Care Medicine, Mayo Clinic, Rochester, USA; 16 Research, WellSpan Health, York, USA

**Keywords:** general nephrology dialysis and transplanation, proliferative glomerulonephritis with monoclonal igg deposits, pgmnid, rapidly progressing glomerulonephritis, rpgn

## Abstract

Proliferative glomerulonephritis with monoclonal immunoglobulin G (IgG) deposits (PGNMID) is a relatively rare diagnosis with variable presentation. When detectable, the disease is typically indolent rather than malignant and recurs in transplant cases. Here, we report a case of PGNMID, which presented clinically as rapidly progressive glomerulonephritis (RPGN). The patient presented to his primary care physician’s office with diarrhea for one day and was admitted for acute kidney injury. Urine sediment was active, and the patient had nephrotic range proteinuria. Serologic workup was negative for any monoclonality: ANA, c-ANCA, and p-ANCA. Kidney biopsy showed diffuse proliferative and crescentic glomerulonephritis with IgG3-kappa restricted deposits, consistent with PGNMID. The patient required dialysis initiation, and corticosteroids were administered. The patient declined further immunomodulatory treatment and remains hemodialysis-dependent. This case highlights the potential for severe renal damage from monoclonal proteins despite an indolent or even undetectable hematologic clone. This entity needs further studies to better understand its immuno-physiological background and develop a standard treatment regimen.

## Introduction

Proliferative glomerulonephritis with monoclonal immunoglobulin G (IgG) deposits (PGNMID) is a rare kidney disease [1]. Its presentation varies, from subclinical to acute nephritic to full nephrotic syndrome, and often progresses in a more insidious fashion. It is usually associated with a monoclonal protein in serum or urine and detectable plasma cell clone (e.g., monoclonal protein of renal significance). The disease results in proteinuria and a slow decline in renal function. There are no specific guidelines regarding the treatment, and various regimens have been tried with variable results [2]. Here, we present a case of PGNMID, which presented as rapidly progressive glomerulonephritis (RPGN) instead of the usual insidious clinical course.

## Case presentation

The patient is a 62-year-old mixed-race male with a past medical history of hypertension, hyperlipidemia, iron deficiency anemia, hypothyroidism, gastric ulcers, 40-pack-year smoking history, and alcohol abuse. At the clinic, he presented to his physician with complaints of multiple episodes of diarrhea for one day. He had blood drawn and was asked to come to the emergency room because of abnormal laboratory findings. The patient was found to have acute kidney injury (AKI). He was initiated on dialysis and underwent an extensive workup, as mentioned below.

Significant laboratory findings included creatinine 13.5 (0.6-1.3 mg/dL), bicarbonate 11 (22-24), CRP 212 (0-2 mg/dL), and calcium 6.8 (8.5-10.0 mg/dL). The free light chain ratio was 1.57 (0.6-1.65). Serum protein electrophoresis demonstrated an acute phase response with low albumin and diffuse polyclonal elevation. Serum immunofixation showed a polyclonal IgA increase. No monoclonal protein was detected.

**Figure 1:**
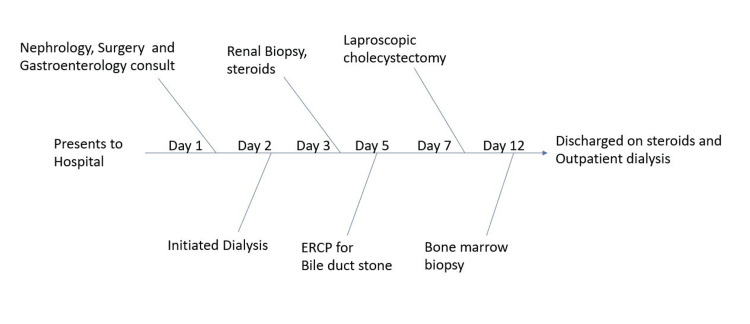
Patient hospital course flow diagram

Simultaneously, during the hospital course (Figure 1), he underwent cholecystectomy for acute choledocholithiasis and developed multifocal atrial tachycardia, which was treated with metoprolol. The patient reported mild chronic bilateral lower extremity swelling of unknown duration that had slightly worsened over the last few days.

Pathologic findings on biopsies

Kidney Biopsy

**Figure 2:**
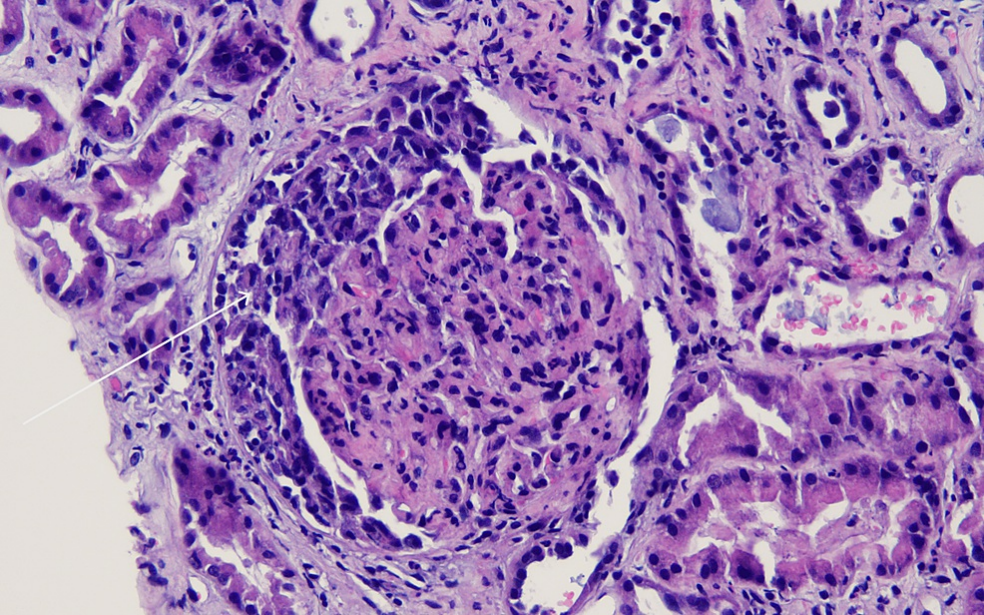
Hematoxylin and eosin stain showing cellular crescent and mesangial hypercellularity

**Figure 3:**
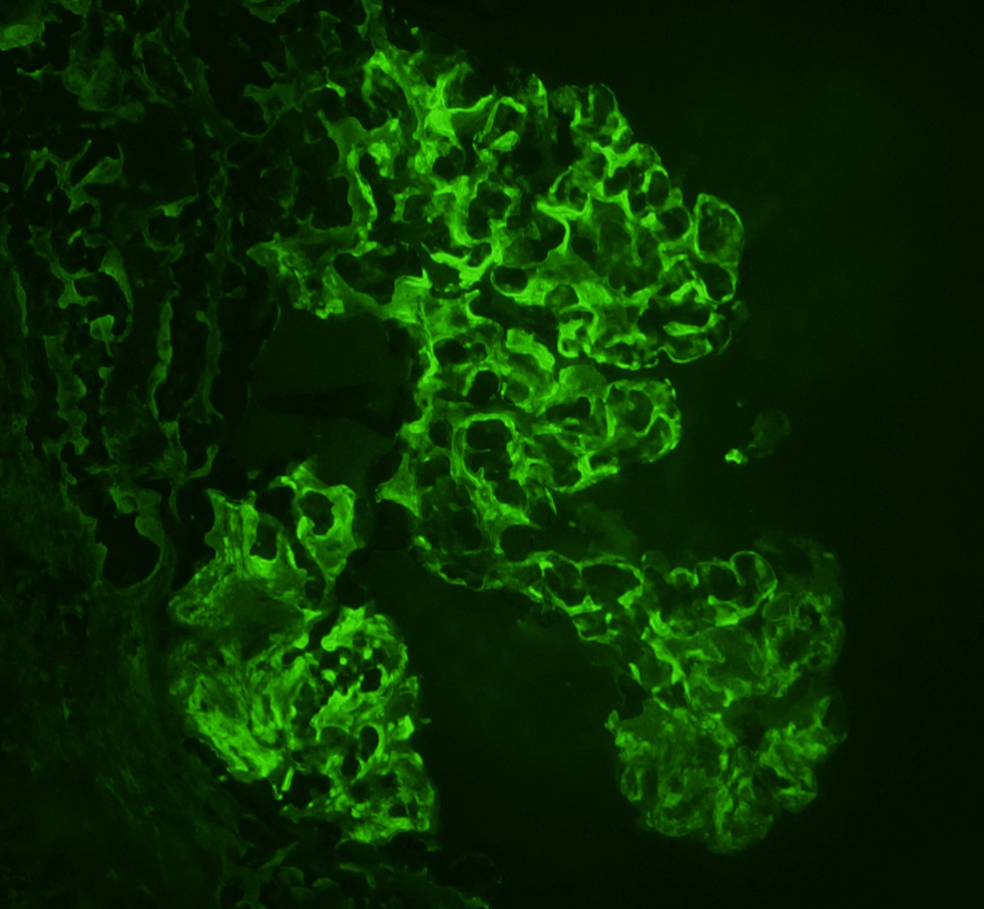
Immunofluorescence showing dense and lobar membranous staining of IgG

**Figure 4:**
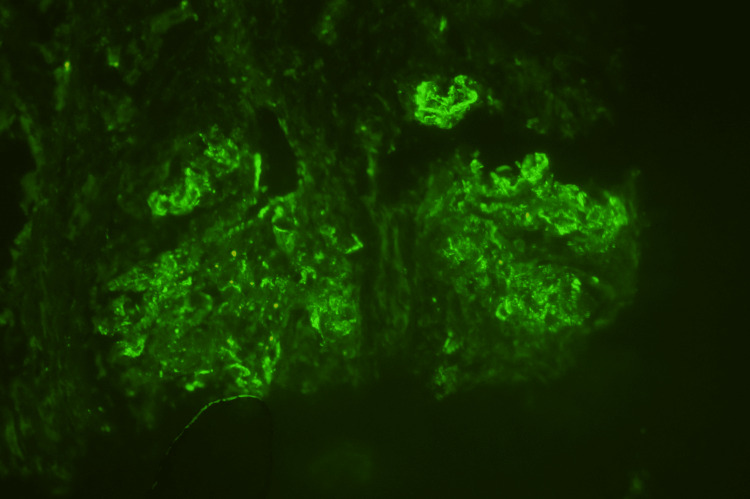
Immunofluorescence showing dense and granular membranous staining of IgG subclass 3

Light microscopy demonstrated diffuse proliferative and crescentic glomerulonephritis (Figure 2). Immunofluorescence showed strong staining for IgG (Figure 3), C3, and kappa light chain but negative staining for lambda light chain and the other heavy chains. Additional IgG subclass staining revealed strong staining for IgG3 (Figure 4), with a trace of negative staining for the other subclasses. Electron microscopy (EM) showed deposits without evident substructure. All of these findings supported the diagnosis of PGNMID. A diagnosis of PGNMID with severe activity and mild chronicity was made.

Bone Marrow Biopsy

Kappa-in situ hybridization (ISH) and lambda-ISH staining were performed with appropriate controls. This showed a polyclonal plasma cell population within the marrow. Congo red staining was negative for amyloid deposition.

The patient was treated with a course of steroids (as used by others in these and overlapping conditions) and prednisone - a dose of 40 mg once a day for the duration of the hospital stay. He responded partially with improvement in urine output and some decline in creatinine/estimated glomerular filtration rate (eGFR) to 4.5/10. He was started on hemodialysis with outpatient follow-up with oncology recommended, which the patient declined. The patient remains dialysis-dependent.

## Discussion

We report a case of PGNMID that presented with a clinical diagnosis of RPGN. The biopsy showed features of diffuse proliferative glomerulonephritis with substantial crescent formation. This case is notable for its substantial crescent formation and RPGN presentation. This is unusual, as this entity often presents in a more indolent clinical and pathologic fashion. The patient was treated with steroids but did not respond to this.

PGNMID is an entity placed under the umbrella of a disease called monoclonal gammopathy of renal significance (MGRS). The entity of MGRS encompasses light chain deposition disease, heavy chain deposition disease, C3 glomerulopathy with monoclonal gammopathy, and light chain proximal tubulopathy, among others.

There is no standard treatment regimen established for the diagnosis [3]. It is mostly treated as MGRS, and both clone-directed chemotherapy and generic immunosuppressive therapy - steroids [2,4], rituximab [5], daratumumab [6], and cyclophosphamide have been tried for both native and transplanted grafts. Response to treatment options like daratumumab, an anti-CD38-targeting antibody that depletes plasma cells and also disturbs humoral immune responses, indicates that more studies and better understanding regarding the pathology of the entity are required in order to develop a protocol-based treatment regimen [7]. Even treatment with plasma exchange has been attempted, but a standard treatment has yet to be identified. Given the lack of standard treatment and wide range of presentation, it could be possible that many patients are underdiagnosed and even under-treated, save for the use of angiotensin-converting enzyme inhibitors in many elderly patients due to their other comorbidities. The disease also has a high recurrence rate after transplant, leading to loss of the graft [8]. As such, conservative treatment with angiotensin-converting enzyme inhibitor has also been implemented in patients with stable creatinine and sub-nephrotic range proteinuria after the disease has occurred in the transplanted graft [9].

There was no evidence of bone marrow or peripheral blood lymphocytic or plasma cell clones in our case. It is important to note that the patient had a contributing comorbidity: septic choledocholithiasis with surgical intervention in the background of alcoholic hepatitis. The patient did have a nonspecific elevation of gamma globulin that could be attributed to both liver disease and acute phase reaction. According to Gumber et al., the rate of clone detection in PGNMID is only about 32% [3]. Immunoglobulin subclass staining by immunofluorescence should be performed when immunofluorescence findings are noted (IgG and light chain) in the absence of electron-dense deposits by EM, especially within the morphologic category of crescentic glomerulonephritis. IgG subclass staining after protease digest on paraffin-fixed material is key and can help confirm the monoclonal nature of the positive IgG staining by immunofluorescence and so the diagnosis of PGNMID.

The rate of response to treatment and outcomes are also variable. Our patient did not receive all the possible treatments due to non-compliance; therefore, he remains dialysis-dependent till the time of writing this report.

## Conclusions

In summary, we presented a case of de novo PGNMID that clinically presented as RPGN, an uncommon presentation of the disease. The disease presents in various forms and, although rare, should not be ruled out in the case of RPGN/crescentic glomerulonephritis.

This case highlights the potential for severe renal damage, possibly from monoclonal proteins, despite an otherwise indolent or even undetectable hematologic clone. More clinical studies are needed to better understand the diagnosis and develop a more standard treatment regimen.
